# Analysis of Microbiota Persistence in Quebec’s Terroir Cheese Using a Metabarcoding Approach

**DOI:** 10.3390/microorganisms10071381

**Published:** 2022-07-09

**Authors:** Annick Raymond-Fleury, Marie-Hélène Lessard, Julien Chamberland, Yves Pouliot, Eric Dugat-Bony, Sylvie L. Turgeon, Daniel St-Gelais, Steve Labrie

**Affiliations:** 1 Department of Food Sciences and Nutrition, Institute of Nutrition and Functional Foods (INAF), STELA Dairy Research Centre, Université Laval, 2425 rue de l’Agriculture, Quebec City, QC G1V 0A6, Canada; annick_raymond@hotmail.com (A.R.-F.); marie-helene.lessard@fsaa.ulaval.ca (M.-H.L.); julien.chamberland@fsaa.ulaval.ca (J.C.); yves.pouliot@fsaa.ulaval.ca (Y.P.); sylvie.turgeon@fsaa.ulaval.ca (S.L.T.); damilk83@hotmail.com (D.S.-G.); 2 UMR SayFood, INRAE, AgroParisTech, Université Paris-Saclay, Avenue Lucien Brétignières, 78850 Thiverval-Grignon, France; eric.dugat-bony@inrae.fr; 3 Agriculture and Agri-Food Canada, Saint-Hyacinthe Research and Development Center, 3600 Casavant Boulevard West, Saint-Hyacinthe, QC J2S 8E3, Canada

**Keywords:** metabarcoding, amplicon sequencing, cheese, Quebec, terroir, fungi, bacteria, microbiota

## Abstract

Environmental short amplicon sequencing, or metabarcoding, is commonly used to characterize the bacterial and fungal microbiota of cheese. Comparisons between different metabarcoding studies are complicated by the use of different gene markers. Here, we systematically compare different metabarcoding molecular targets using V3–V4 and V6–V8 regions of the bacterial 16S rDNA and fungal ITS1 and ITS2 regions. Taxonomic profiles varied depending on the molecular markers used. Based on data quality and detection capacity of the markers toward microorganisms usually associated with the dairy environment, the ribosomal regions V3–V4 and ITS2 were selected and further used to evaluate variability in the microbial ecosystem of terroir cheeses from the province of Quebec in Canada. Both fungal and bacterial ecosystem profiles were described for 32 different ready-to-eat bloomy-, washed- and natural-rind specialty cheese varieties. Among them, 15 were studied over two different production years. Using the Bray–Curtis dissimilarity index as an indicator of microbial shifts, we found that most variations could be explained by either a voluntary change in starter or ripening culture composition, or by changes in the cheesemaking technology. Overall, our results suggest the persistence of the microbiota between the two years studied—these data aid understanding of cheese microbiota composition and persistence during cheese ripening.

## 1. Introduction

Variations in cheese quality may be explained by fluctuations in milk composition [1,2] that affect coagulation properties [3], curd drainage [3] and yield [4]. Fluctuations in the activity of complex microbial cheese ecosystems can also influence product consistency [5,6,7]. Bacteria and fungi are responsible for the typical organoleptic properties of cheese. Characterization of the bacteria and fungi contained in cheese can therefore help reduce variations in cheese quality and reduce potential associated economic losses [8].

In 2012, over 600 different yeast and mold species were isolated from raw milk produced in Quebec and destined for cheesemaking [9], highlighting that fungi naturally present in milk can develop in cheese, potentially contributing to ripening. However, this study did not analyze bacteria or use the deep characterization that high-throughput sequencing (HTS) methods now allow. The decreasing cost of HTS profiling methods provides an unprecedented opportunity to describe and/or monitor complex microbial communities. Many cheese ecosystems from different countries have been characterized using such HTS technologies [6,10,11,12]. This has allowed the detection of new microorganisms not previously associated with cheeses and emphasized the importance of the so-called secondary microbiota, including non-inoculated microbiota that develops during the late stages of ripening [5]. HTS methods such as high-throughput amplicon-based metagenomics, or metabarcoding, have made it possible to identify the dominant and subdominant microorganisms involved in an ecosystem, whether they are cultivable in the laboratory or not [13].

Metabarcoding is performed using phylogenetic markers such as the nine highly variable regions of the 16S rDNA (V1–V9) [6,11], the fungal internal transcribed spacers (ITS1 and ITS2) [14,15,16], and the 18S rDNA or the D1/D2 domain of the 26S rDNA [17]. To date, no consensus has been made about the best metabarcoding targets for the cheese ecosystem, even though they greatly influence the resulting microbial profiles [18,19]. Target region, amplicon length and possible primer mismatches have a direct impact on taxonomic assignment, therefore influencing the measured composition of the same microbial community [20,21]. For example, primer mismatches may induce a distorted taxonomic assessment due to the inability to detect certain groups or genera and the subsequent overestimation of others [22].

Dairy microbiota have previously been studied using the hypervariable regions of the bacterial 16S rDNA V1–V3 [10,23,24], V3–V4 [11,25,26], V4 [5,6,7], V5–V6 [13] and V6–V8 [27], and of the fungal ITS1 [6,7,12] and ITS2 [11,28]. Regions V3–V4 or V4 are used more frequently to characterize cheese ecosystems compared to V6–V8. Both ITS regions (ITS1 and ITS2) provided similar results for extended studies but yielded different taxonomic resolutions and genera detections according to the environment studied [18,19,21]. It is, therefore, important to compare markers for specific ecosystems.

To monitor the bacterial and fungal microbiota of terroir cheese from Quebec, we first compared the performance of primers targeting two bacterial 16S rDNA regions (V3–V4 and V6–V8) and two fungal ITS regions (ITS1 and ITS2). We identified V3–V4 and ITS2 as the most appropriate molecular markers to characterize bacterial and fungal ecosystems, respectively, in bloomy-, natural- and washed-ripened specialty cheeses (soft, semi-hard and hard varieties). We evaluate the consistency of the microbial ecosystems in Quebec’s terroir cheese by comparing production years 2015 and 2018. This article reports the first evaluation of the microbial diversity of Quebec’s terroir cheese ecosystems and their variability across different production years.

## 2. Materials and Methods

**Cheese sampling.** A total of 47 ready-to-eat terroir cheeses from Quebec were sampled and analyzed (Table S1). Twenty-seven cheeses made in 2015 were purchased in a single copy from a specialized shop in Quebec City (Québec, QC, Canada). Twenty cheeses produced in 2018 were received in triplicate directly from the cheesemakers (Table S1). These 47 cheeses belong to 32 cheese varieties (15 cheeses from 2015 and 2018, 12 cheeses only from 2015 and 5 varieties only from 2018). Samples included bloomy-, washed- or natural-rind cheeses, the latter being washed with a diluted salt solution but without the addition of ripening starters.

The core (10 g) and rind (25 cm^2^) cheeses were sampled with sterile scalpels and analyzed separately (98 samples in total). Cryogenic grinding was performed using the CryoMill apparatus (Restch^®^, Haan, Germany) with liquid nitrogen to obtain a fine frozen cheese powder. The grinding step consisted of an automatic precooling step (>3 min) at a 5 Hz frequency followed by two cycles at 25 Hz for 2 min and a 30-sec cooling step at 5 Hz between the cycles. The samples were named according to the section of the cheese analyzed (c for center, r for rind) and the year of the sampling (e.g., c01y15; center of cheese 01, year 2015).

**DNA extraction, library construction and sequencing.** DNA extraction was performed using the PureLink^TM^ Viral RNA/DNA Mini Kit (Invitrogen, Life Technologies, Carlsbad, CA, USA), according to the manufacturer’s protocol, with minor modifications. A 30–40 mg sample of ground cheese was homogenized in 200 µL of NaCl 0.9% before proteinase K and the lysis buffer were added, following the Purelink protocol. DNA concentration and quality were determined using the Nanodrop (ND-1000) spectrophotometer (ThermoFisher Scientific, Wilmington, NC, USA).

Targeted amplification from total genomic DNA, metabarcoding library construction using specific rDNA primers (Table S2), equimolar pooling, MiSeq high-throughput sequencing (Illumina, San Diego, CA, USA) and paired-end reads (2 × 300 bp) demultiplexing was performed at the Institut de Biologie Intégrative et des Systèmes (IBIS, Université Laval). The bacterial and fungal composition of the core and the rind were determined by targeting the rDNA 16S region V3–V4, respectively [29], or V6–V8 [30] and the Internal Transcribed Spacer 1 or 2 (ITS1, ITS2) [31].

**Bioinformatic analysis, metrics and figures.** Metabarcoding analysis was performed using FROGS with most default parameters [32,33]. Briefly, paired-end sequences were clustered using the swarm clustering method [34], with a maximum of 3 differences (d = 3) allowed between amplicons. All clusters were filtered for chimeras using VSEARCH [35]. Operational Taxonomic Units (OTUs) with an abundance inferior to 0.005% were discarded because they are more likely to be chimeras than rare clusters [36] and may lead to a false overestimation of sample richness. Finally, the affiliation of all bacterial OTUs was validated using the EzBioCloud database [37] and the sequences from type material in 16S rRNA and ITS databases for BlastN in NCBI [38]. The remaining chimeras identified were manually filtered, and these cured OTU tables were used to calculate the biostatistics using PhyloSeq R package v1.28.0, implemented in FROGS [39]. Alpha-diversity metrics (Chao1 and inverse Simpson indexes) were computed after rarefaction. The abundance-based dissimilarity (beta-diversity) index, the Bray–Curtis index [40], was also calculated after rarefaction. Histograms presented in this study were drawn using the R package ggplot2 v.2.2.1 library [41].

## 3. Results

### 3.1. Sequencing Data and Taxonomic Assignation

rDNA libraries were prepared separately for the core and the rind from a total of 32 different ready-to-eat cheeses (Table S1). Two different DNA targets were used for bacteria (V3–V4 and V6–V8 regions from the rDNA 16S) and fungi (ITS1 and ITS2 of the rDNA ITS region) to compare their ability to characterize cheese microbiota using different primer sets (Table S2). A total of 21,616,072 sequence reads were obtained through the Illumina MiSeq sequencing platform.

For the bacterial profiles, 4,505,810 and 6,156,446 paired-end reads were sequenced for the V3–V4 and V6–V8 regions, respectively. Overall, 2,588,028 and 3,451,507 non-chimeric reads were assigned, respectively, to 108 and 154 OTUs, having a relative abundance >0.005% (Table 1). The rDNA target V3–V4 allowed the identification of 57 genera, 22 of which were highly abundant (>10% relative abundance in one or more samples). The V6–V8 region allowed the identification of 82 genera, including 21 highly abundant genera. In total, 38 bacterial genera were detected by both 16S rDNA targets (Table 1).

For the fungal profiles, 5,207,694 and 5,746,122 reads were sequenced for intergenic regions ITS1 and ITS2, respectively. Of this total, 4,683,355 and 5,096,678 non-chimeric sequences were assigned to 67 and 78 OTUs (>0.005%), respectively (Table 1). Among these OTUs, ITS1 and ITS2 allowed the identification of 31 and 34 fungal genera, including 18 genera identified using both regions (Table 1).

### 3.2. Choice of the Best Targets for Metabarcoding

**Figure 1:**
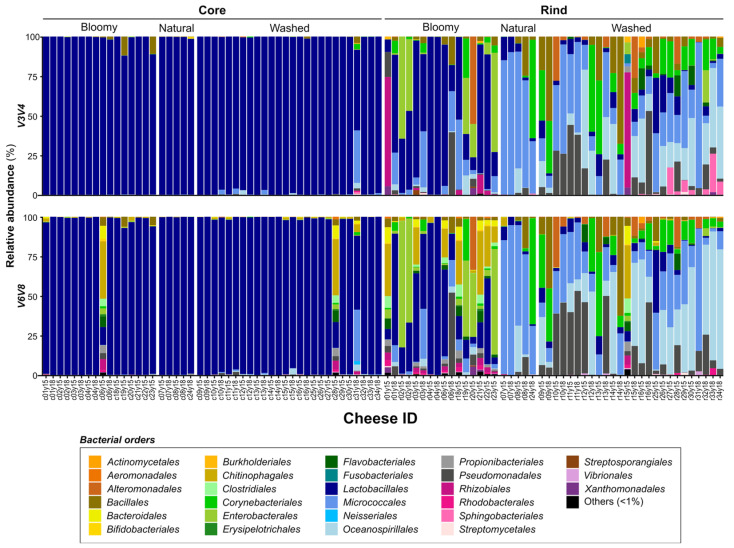
**Comparative distribution of the most abundant bacteria in cheese ecosystems.** Each column shows the relative abundance of the bacterial microbiota (16S rDNA), representing over 1% of the average abundance (order <1% are combined and shown in black). Vertical sections show different 16S DNA targets: the top row shows V3–V4 data, and the bottom row shows V6–V8 data. Horizontal sections show different parts of the cheese according to the rind type (bloomy, natural or washed): the left row shows cheese core data, and the right row shows cheese rind data. The last number of the cheese ID refers to the year of production (2015 or 2018) for the 32 cheese varieties.

A comparison of different rDNA regions for monitoring the bacterial microbiota in cheese provided similar profiles (Figure 1). At the order taxonomic level, the major taxa identified were *Lactobacillales* (70%), *Micrococcales* (8–10%), *Oceanospirillales*, *Pseudomonadales*, *Enterobacterales*, *Corynebacteriales*, *Bacillales* and *Rhizobiales* (Dataset S1). For bacteria, V3–V4 generated fewer chimeric sequences and fewer low-abundant OTUs than V6–V8 (33.7% compared to 37.0%) (Table 1). In addition, V6–V8 provided ten ambiguous profiles out of the 94 samples sequenced (c06y15, c28y15, r01y15, r03y15, r06y15, r18y15, r21y15, r22y15, r23y15, r15y15; see Section 2 for the sample nomenclature) that could be associated with background or unspecific sequencing. In contrast, only two ambiguous profiles were present using V3–V4 (Figure 1; r01y15, r15y15), therefore suggesting that V3–V4 was a better bacterial target for metabarcoding studies in cheese ecosystems. The two samples suspected to correspond to background or unspecific amplification, namely r01y15 and r15y15, were removed for further analysis.

**Figure 2:**
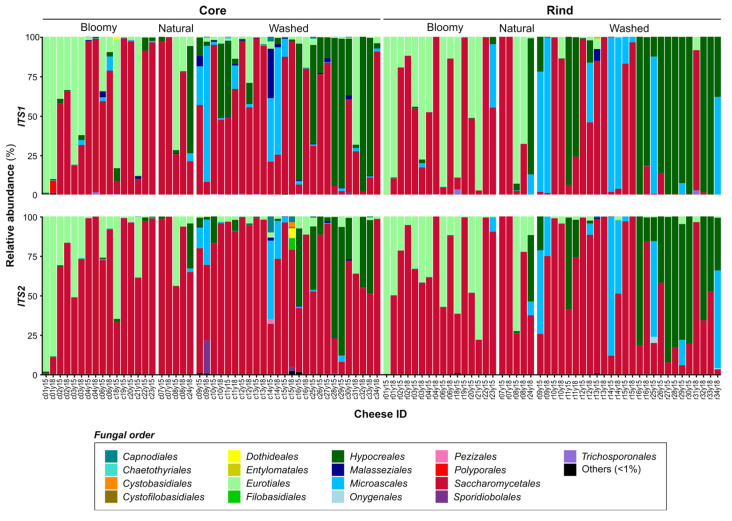
**Comparative distribution of the most abundant fungi in cheese ecosystems.** Each column shows the relative abundance of the fungal (ITS) microbiota representing over 1% of the average abundance (order <1% are combined and shown in black). Vertical sections show different ITS targets: the top row shows ITS1 data, and the bottom row shows ITS2 data. Horizontal sections show different parts of the cheese according to the rind type (bloomy, natural or washed): the left row shows cheese core data, and the right row shows cheese rind data. The last number of the cheese ID refers to the year of production (2015 or 2018) for the 32 cheese varieties.

Fungal metabarcoding performed using ITS1 and ITS2 regions (Figure 2) generated a low proportion of chimeric sequences and rare OTUs, corresponding to 5.0% and 10.2% of the sequences, respectively (Table 1). The fungal profiles were different between ITS1 and ITS2. For ITS1, *Saccharomycetales* (52%), *Hypocreales* (21%), *Eurotiales* (17%) and *Microascales* (9%) were detected as the most abundant orders, while in ITS2 profiles, most sequence reads were affiliated to *Saccharomycetales* (73%), *Eurotiales* (12%), *Hypocreales* (10%) and *Microascales* (4%) (Dataset S1). The large dominance of *Saccharomycetales* using ITS2 is in accordance with specific dairy fungi and supports other evidence indicating that it is the most appropriate target for the characterization of fungal communities in cheeses [31].

### 3.3. Microbial Landscape of Quebec’s Terroir Cheeses

**Figure 3:**
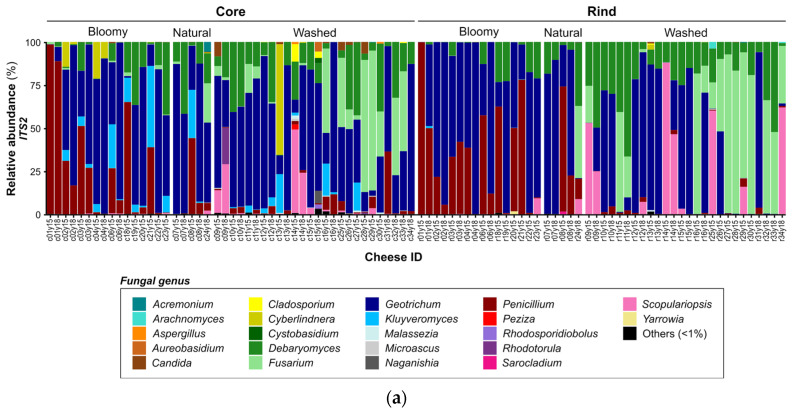

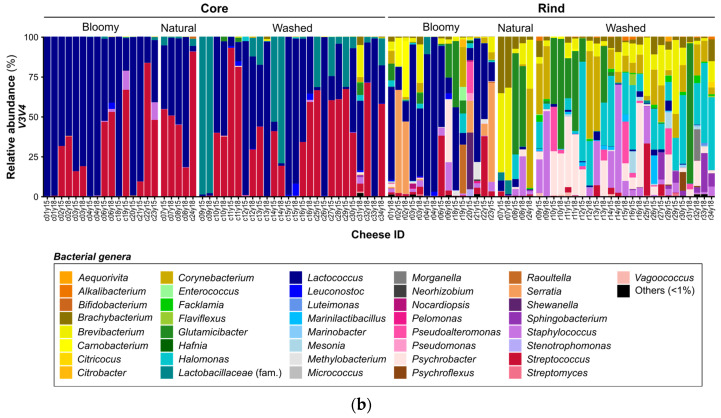
**Comparative distribution of the most abundant genera in cheese ecosystems.** Horizontal sections show different parts of the cheese according to the rind type (bloomy, natural or washed): the left row shows cheese core data, and the right row shows cheese rind data. The last number of the cheese ID refers to the year of production (2015 or 2018) for the 32 cheese varieties. (**a**) Each column shows the relative abundance of the bacterial (V3–V4) microbiota representing over 1% of the average abundance (order <1% are combined and shown in black). (**b**) Each column shows the relative abundance of the fungal (ITS2) microbiota representing over 1% of the average abundance (order <1% are combined and shown in black).

Cheese core ecosystems were largely dominated by starter and ripening cultures [11]. Overall, these cheese cores ecosystems were composed of OTUs affiliated with 47 bacterial genera where the most abundant were *Lactococcus* (54%), *Streptococcus* (37%) and the *Lactobacillaceae* family (7%), the latter being present mostly in washed cheese cores (Dataset S1 and Figure 3a). The fungal OTUs were mostly affiliated with the genera *Geotrichum*, *Debaryomyces*, *Penicillium*, *Fusarium*, *Kluyveromyces*, *Cyberlindnera* and *Scopulariopsis* (Dataset S1 and Figure 3b). Fungal core ecosystems showed specific profiles related to rind type. *Geotrichum* (46%) and *Debaryomyces* (20%) were the most abundant yeasts in washed cheese cores, while the surface of the bloomy-rind cheeses was dominated by *Geotrichum* (52%) and *Penicillium* (28%) (Dataset S1 and Figure 3b).

α-diversity was assessed for all cheese samples studied (Table 2). The Chao index evaluates richness by estimating the number of OTUs in a community [42], and the inverse of the Simpson index is an α-diversity measure (within sample diversity) based on the number of OTU and their abundance [43]. In cheese cores, both the average richness and diversity indexes were similar for fungi and for bacteria, regardless of rind type. The maximum estimated OTU number reached 30 for fungi and 42 for bacteria, while the maximum inverse of the Simpson α-diversity index reached 3.183 and 2.516 for both kingdoms, respectively (Table 2, Table S1).

Cheese rind ecosystems were dominated by lactic acid bacteria (LAB), ripening strains and members of the secondary microbiota found in cheeses. On the surface of the cheese sampled, the average fungal richness (18 OTUs) and inverse Simpson index (1.919) were lower than the bacterial richness (34 OTUs) and inverse Simpson index (3.601), no matter the type of rind (Table 2). Overall, the cheese rind’s ecosystems were composed of OTUs affiliated to 54 bacterial genera where the most abundant were *Lactococcus*, *Brevibacterium*, *Glutamicibacter*, *Corynebacterium*, *Halomonas*, *Brachybacterium*, *Staphylococcus*, *Psychrobacter*, *Serratia*, *Streptococcus*, *Pseudoalteromonas* and several other less abundant genera (Figure 3a, Dataset S1). The fungal OTUs were mostly affiliated with the genera *Geotrichum*, *Penicillium*, *Debaryomyces*, *Fusarium* and *Scopulariopsis* (Figure 3b, Dataset S1).

These rind ecosystems showed, at the genus taxonomic level, specific populations depending on the type of rind. The highest bacterial richness and α-diversity were observed in washed rinds (maximum richness of 46 OTUs and inverse Simpson index of 7.268), followed by bloomy and natural rinds (Table 2). The richness and inverse Simpson index of fungal cheese rinds were similar in all rinds and to those observed in cheese cores (Table 2).

Washed rinds were dominated by halotolerant psychrophilic and coryneform bacteria belonging to the genera *Halomonas* (20%), *Corynebacterium* (13%), *Glutamicibacter* (13%), *Psychrobacter* (12%), *Staphylococcus* (10%) and *Brevibacterium* (10%) (Figure 3a, Dataset S1). Their fungal ecosystem was mostly composed of *Geotrichum* (35%), *Fusarium* (28%), *Debaryomyces* (22%) and *Scopulariopsis* (14%) (Figure 3b, Dataset S1).

Bloomy rinds showed a different microbiota composed mostly of the fungi *Geotrichum* (58%), *Penicillium* (34%) and *Debaryomyces* (7%), and the bacteria *Lactococcus* (44%), *Serratia* (13%), *Streptococcus* (6%), *Glutamicibacter* (6%) and many less abundant genera (Figure 3a, Dataset S1).

### 3.4. Assessing the Stability of Quebec’s Terroir Cheese Ecosystems

**Figure 4:**
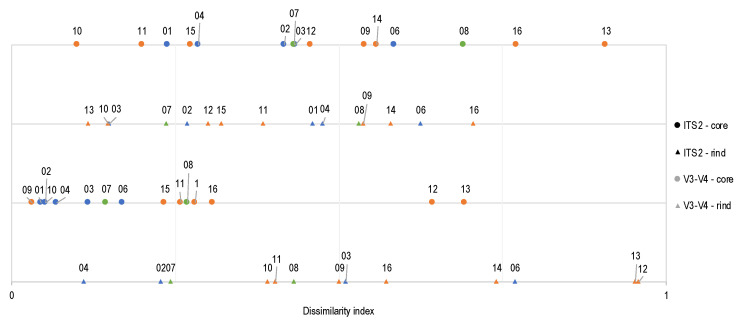
**Similarity of cheese microbiota from different years according to the Bray–Curtis dissimilarity index.** The Bray–Curtis index shows the similarity (0–0.75) or dissimilarity (0.76–1) of the cheeses from different productions (2015 and 2018). The index has been calculated for fungi (ITS2) and bacteria (V3–V4) from cheese rind (▲) and cheese core (○). Numbers shown are cheese identification numbers for the bloomy- (blue), natural- (green) and washed-rind cheeses (orange).

The bacterial (V3–V4) and fungal (ITS2) constancy of cheese ecosystems were evaluated by calculating the β-diversity of 15 cheeses produced in 2015 and in 2018. The β-diversity, which is the diversity observed between samples, was calculated separately for the cheese rinds and cores using the Bray–Curtis dissimilarity index [40] to compare these ecosystems between the two years studied. A 0.5 threshold value was set to distinguish similar (0.0–0.5) from dissimilar (0.5–1.0) communities (Figure 4, Table S1). Because it seemed that background or unspecific sequences were obtained for samples r01y15 and r15y15, they were not included in the comparisons. A total of 58 comparisons were considered.

More than half of the Bray–Curtis dissimilarity index calculated between the cheeses sampled in 2015 and 2018 (~65%) could be interpreted as similar ecosystems. Indeed, for the core ecosystems, 9/15 fungi and 13/15 bacteria, while 10/15 fungi and 6/13 bacteria of the rind ecosystems show a Bray–Curtis β-diversity index <0.5. A few ecosystems tend to present a greater dissimilarity. A total of three cheeses had a Bray–Curtis index >0.9 (ITS2c13, V3–V4r12 and V3–V4r13), and two cheeses had a Bray–Curtis index between 0.75 and 0.89 (ITS2c16 and V3–V4r06) (Figure 4, Table S1).

For the fungi monitored using the ITS2 region, two washed-rind cheese cores (c13 and c16) were among the most dissimilar ecosystems. The changes observed involved only a few yeast and mold genera. The most dissimilar fungal core ecosystem (Bray–Curtis index of 0.908) was observed in a washed-rind cheese core (c13), where proportions of *Cyberlindnera*, *Kluyveromyces* and *Geotrichum* changed from 64%, 22% and 11% to 84%, 14% and 2% respectively, from 2015 to 2018 (Figure 3b, Dataset S1). For c16 (Bray–Curtis 0.772), the major yeast genera found in 2015 were *Fusarium* (49%), *Kluyveromyces* (19%), *Geotrichum* (18%), *Penicillium* (8%) and *Debaryomyces* (3%). In 2018, these proportions switched to *Geotrichum* (87%), *Penicillium* (12%) and *Kluyveromyces* (1%) (Figure 3b, Dataset S1).

High dissimilarities in the bacterial ecosystems were observed on the surface of two washed-rind cheeses (r12 and r13) and one bloomy cheese (r06). For the bloomy cheese rind (r06), the Bray–Curtis index was 0.769, explained by the high abundance of the starter cultures *Lactococcus* (51%) and *Streptococcus* (38%) in 2015 and by halotolerant psychrophilic bacteria (*Psychrobacter*, *Glutamicibacter* and *Staphylococcus*; totaling 79%) in 2018 (Figure 3a, Dataset S1). The dissimilarities of the washed-rind cheeses involved mostly changes in proportions of the halotolerant psychrophilic bacteria and coryneform bacteria such as *Halomonas*, *Psychrobacter*, *Glutamicibacter*, *Corynebacterium*, *Brevibacterium* and *Staphylococcus* (Figure 3a, Dataset S1).

## 4. Discussion

**Optimal target choice for metabarcoding.** MiSeq sequencing technology was used in this study to analyze Quebec’s specialty cheese microbiota composition. We first compared different molecular markers targeting bacteria (regions V3–V4 and V6–V8 of 16S rDNA) and fungi (intergenic regions ITS1 and ITS2). The accuracy of cheese microbial profiling of both regions targeted in rDNA 16S and ITS was evaluated based on several criteria. Overall, the total number of genera detected and the proportions of dominant genera (relative abundance > 10%) were very similar (Table 1). For bacteria, V3–V4 seems to generate 34% of chimeric sequences and low-abundant OTUs, compared to 37% of chimeric sequences for V6–V8 (Table 1). The chimera sequence rate is higher for bacteria than fungi, perhaps because 16S rDNA is highly conserved, promoting recombination during the PCR amplification step prior to library preparation [44,45]. Despite comparable results for V3–V4 and V6–V8 with other cheese metabarcoding studies [5,6], a lower rate of chimeric sequences favors V3–V4 as a preferable target for bacterial metabarcoding. Moreover, V6–V8 gave 10 samples showing only background amplification profiles, compared to 2 samples for V3–V4 (Figure 1) [46,47]. Aside from possible technical problems in the library preparation, the high number of bloomy-rind cheese rind samples without relevant metabarcoding profiles could be explained by the generally low abundance of subdominant bacteria on the surface of bloomy-rind cheeses, which would have to be verified using a quantification method such as qPCR [7,32,48].

Fungal metabarcoding using ITS1 and ITS2 generated, respectively, 5% and 10% chimeric sequences and low abundant OTUs (Table 1). However, taxonomic assignment favored ITS2 as the best target due to its ability, contrarily to ITS1, to detect genera previously associated with dairy ecosystems such as *Cyberlindnera*, *Cryptococcus*, *Kazachstania*, *Pichia* and *Yarrowia* [9]. Moreover, a high number of polymorphisms have been described previously in *Geotrichum candidum* rDNA, specifically in the ITS1 region [49]. This observation, combined with the fact that the primers used for ITS1 amplification might not allow the detection of the taxonomic class *Saccharomycetes* due to 3′ terminal mismatches with several other fungal genera [31,50,51], leads to the conclusion that ITS2 could provide a more accurate representation of the fungal microbiota for cheeses and the dairy environment [52,53].

**The microbial landscape of Quebec’s terroir cheeses.** For the first time, the bacterial and fungal microbiota of Quebec’s terroir cheeses were studied using metabarcoding. Cheese rinds and cores were analyzed separately because of their different microbial composition [54,55,56]. Due to their contact with the external environment, cheese rind ecosystems generally have a higher α-diversity than cores [57,58]. Here, when comparing ecosystems, the bacterial α-diversity (calculated using Chao1 and inverse Simpson indexes) was generally higher in rinds than in cores, whereas these indexes were similar for fungal populations [11]. Moreover, cheeses cores and rinds showed a higher α-diversity for washed-ripened cheeses than natural- and bloomy-ripened cheeses (Table 2, Table S1).

The predominant bacteria found in cheese cores belonged to the genera *Lactococcus*, *Streptococcus* and the *Lactobacillaceae* family (Figure 3a, Dataset S1). These lactic acid bacteria are commonly used as a starter or adjunct cultures and were found in similar relative abundances for natural- and washed-rind technologies (Figure 3a). In the cheese samples studied, *Lactobacillaceae* were mostly present in washed-rind cheese cores (19%). They might have been added as adjunct cultures for their sugar metabolism and autolytic and proteolytic capacities that change the texture and provide cheese flavor enhancements or as a starter in some hard cheese varieties [59]. They did not seem to be used either as starter or adjunct cultures in bloomy-rind soft cheeses [60,61]. In these samples, *Lactococcus* and *Streptococcus* were observed in various proportions. Soft bloomy cheeses can be produced using either traditional or stabilized cheesemaking technology, the latter requiring the use of thermophilic starters [62,63].

Cheese surface ecosystems are known to have specific microbial compositions depending on cheesemaking technology [5,6,11]. Bloomy rinds are mostly colonized by yeasts and molds [64], and the bacterial microbiota is generally not considered [6]. The high abundance of *Lactococcus* and other bacteria in the bloomy-cheese rinds analyzed may be explained by an inadvertent sampling of the cheese subsurface [65]. This would need additional and complementary characterization, such as qPCR quantification, to provide a more realistic profile of the surface bacterial ecosystems [32]. Washed rind cheese surfaces have been described abundantly in the literature. As other authors found, the washed-ripened cheese ecosystems contained coryneform bacteria (*Brachybacterium*, *Brevibacterium*, *Corynebacterium* and *Glutamicibacter*) and staphylococci, which were important contributors to the typicity of several washed-rind cheeses [66,67,68]. They may have been introduced through the personal and the indigenous ecosystem established within the cheesemaking plants [7,69]. As revealed by other studies, despite the common addition of smear bacteria starters in washed-ripened cheese (*Brevibacterium, Glutamicibacter*), the cheese rind microbiota was rather composed of environmental Actinobacteria (*Corynebacterium*, *Brachybacterium*) and Gammaproteobacteria (*Halomonas*, *Psychrobacter*, *Pseudoalteromonas*). Their occurrence and relative proportions are known to be influenced by many factors such as salt concentration, pH and low temperature of ripened cheese [70,71,72]. Halotolerant psychrophilic bacteria are most likely introduced in cheese ecosystems through the brine, tools and surfaces rather than in raw milk [5,73,74,75,76].

Fungal communities of cheese cores and rinds showed similar composition for a given variety and were mainly composed of *Geotrichum*, *Penicillium*, *Debaryomyces, Fusarium*, *Kluyveromyces*, *Scopulariopsis* and *Cyberlindnera* (Dataset S1). These dairy yeasts and molds frequently occur in cheeses because they are inoculated as ripening cultures or originate from the indigenous microbiota of the milk or of the dairy plant environment [73,77,78,79]. They were found in various proportions, without obvious predominance in either rind type, except for *Penicillium*, which was more abundant in bloomy-rind cheeses and was well known to contribute to the development of their characteristic appearance and flavor (Figure 3b) [78]. In the washed-rind cheese ecosystems, *Fusarium* (28%) was the second most abundant fungi identified in this study (Dataset S1). Although most commonly found in bloomy-rind cheeses [65], *Fusarium* can be added to the smearing solutions to reduce rind stickiness [80]. *Scopulariopsis* (14%, Dataset S1), usually associated with wrapped washed- and natural-rind cheeses [81], can contribute to cheese flavor through ammonia production [82]. Although generally considered spoilage molds [83,84,85], *Fusarium* and *Scopulariopsis* could have been used as ripening agents for their technological properties [78].

The metabarcoding method used in this study allowed the detection of the major contributors to cheese ecosystems and the influence of technological practices, such as the choice of optimal ripening and adjunct cultures, on each cheese variety studied (bloomy-, natural- and washed-rind). These data also provide information on the natural microbiota that could develop during cheese ripening. However, using metabarcoding does not give a quantitative overview of the microbiota nor its impact on organoleptic properties. Further studies using complementary techniques, such as qPCR, texture profile analysis, proteolysis and lipolysis assessment, would be necessary to give a complete overview of these cheeses.

**Assessing the stability of Quebec’s terroir cheese ecosystems.** This study analyzed the same cheese varieties (numbered 01 to 16) produced in different years for the first time. The persistence of the microbial communities was thus evaluated using the Bray–Curtis distance [40]. This dissimilarity index, which is a reliable index to point out changed environments, was calculated for 58 comparisons (bacteria and fungi located in the core or on the rind) based on abundance data [86]. Most cheeses showed high similarity of the microbiota from year to year. This may be explained in part by the high abundance of the starter and ripening cultures inoculated during the cheesemaking process. However, the optimized metabarcoding method is sensitive enough to monitor changes in some ecosystems (cheeses 06, 12, 13 and 16). Cheese ecosystems can be modulated by numerous factors, including variation in the indigenous microbiota composition of the milk, the use of heat treatment [5,87], the cheesemaking process (starter, ripening and adjunct cultures used, smearing solutions, brining, utensils and staff) [6,65,76] and the processing/ripening conditions (shelves, temperature, relative humidity, cheese packaging, ripening time and air composition) [1,5,6,7,65,88,89].

The highest variations between the two years studied were observed in the cheese ecosystems ITS2c13, V3–V4r12 and V3–V4r13, as indicated by a Bray–Curtis index >0.9 (Figure 4). The most dissimilar rind ecosystems were the washed-rind cheeses 12 and 13 (Bray–Curtis indexes 0.958 and 0.952, respectively). The surface of the washed-rind cheeses had a high bacterial α-diversity [6,11,67,77], and the dissimilarity observed between 2015 and 2018 was characterized by shifts in the relative abundance of halotolerant psychrophilic Gammaproteobacteria (*Pseudoalteromonas*, *Halomonas* and *Psychrobacter*), which, to our knowledge, are not added to cheese as ripening starters. The shifts observed between 2015 and 2018 were possibly influenced by a change in the humidity content of the cheese rind [6], affected by possible variations in the environment of the ripening chambers (temperature, relative humidity and air velocity), the salinity of the washing (smear) solution used [75,76,90], the quality and renewal frequency of brine solutions [76,90,91], the environment in the ripening chamber and/or of the cheesemaking plant [7].

The changes monitored in the dissimilar fungal core ecosystem of cheese 16 (Bray–Curtis index 0.908) were mostly explained by the addition of ripening cultures. The fungal ecosystem described for the 2015 production of cheese 16 was mostly composed of *Cyberlindnera* (64%), *Kluyveromyces* (22%) and *Geotrichum* (11%), which could have been deliberately added as starters culture or could originate from the indigenous milk microflora [92]. *Cyberlindnera* is a facultative fermenting yeast rarely documented in dairy products and mostly found in cheese cores (Figure 3b) [93]. Its occurrence in Quebec’s terroir milk and cheese have been previously described using traditional microbiology [9] and could be explained by milk or brine contamination [94]. *Cyberlindera* produces ethyl acetate and acetaldehyde that could contribute to the cheese flavor [95]. In 2018, the core of the same cheese was largely dominated by the fungal starters *Geotrichum* and *Debaryomyces*, two yeasts frequently inoculated in washed-rind cheeses for their technological properties [77]. *Debaryomyces* can also be found in cheese from involuntary inoculation since it is widespread in the cheesemaking plant environment, especially in brine, due to its tolerance to high salt concentrations and low temperature [7,76,96,97]. These yeasts are therefore highly abundant in cheese cores.

## 5. Conclusions

In conclusion, metabarcoding provided for the first time the microbial profile of many different bloomy-, washed- and natural-rind terroir cheeses from Quebec, evaluating the richness and diversity of both the cores and rinds. This large-scale metabarcoding inventory proposes that V3–V4 and ITS2 are adequate molecular identifiers to explore cheese microbial diversity in depth. This approach leads to an accurate identification (genus-level) within a few hours of analysis using FROGS and allows an understanding of the dissimilarities observed in cheeses through time. This study highlights the persistence of key bacteria and fungi in the Quebec terroir cheeses. More in-depth microbiological analysis could provide biological indicators to help keep high-quality products and reduce specialty cheese spoilage. The level of metabolic activities or quantitative determination of the microbial load was not evaluated and should be pursued in the future.

## Figures and Tables

**Table 1:** Sequencing data of the fungal (ITS1 and ITS2) and the bacterial (V3–V4 and V6–V8) ecosystems.

Target Region	Bacteria		Fungi	
	V3–V4	V6–V8	ITS1	ITS2
Nb. reads sequenced ^1^	4,505,810	6,156,446	5,207,694	5,746,122
Nb. trimmed and assembled sequences	3,904,825	5,482,054	4,931,241	5,675,978
Nb. non-chimeric OTU sequences ^1^	2,588,028	3,451,507	4,683,355	5,096,678
Non-chimeric sequences	66.28%	62.96%	94.97%	89.79%
Nb. non-chimeric OTUs ^1^	108	154	67	78
Nb. genera assigned ^1^/Nb. shared genera assignation	57/ 37	83/ 37	34/ 18	35/ 18
Nb. abundant genera ^2^/Nb. shared genera assignation	22/ 17	20/ 17	9/ 6	9/ 6

^1^ with relative abundance >0.005%. ^2^ abundant genera with relative abundance >10%.

**Table 2:** Richness and α-diversity of cheese microbiota.

Cheese Section	Rind Type	Bacteria		Fungi	
		Richness ^1^	α-Diversity ^1^	Richness	α-Diversity
Cheese core	Bloomy	16 ± 11	1.497 ± 0.517	23 ± 6	2.020 ± 0.829
	Natural	16 ± 7	1.783 ± 0.430	21 ± 4	2.251 ± 0.932
	Washed	24 ± 18	1.794 ± 0.722	24 ± 6	2.317 ± 0.800
Cheese rind	Bloomy	32 ± 8	3.026 ± 1.683	19 ± 10	1.745 ± 0.496
	Natural	34 ± 16	2.732 ± 0.372	18 ± 10	2.039 ± 1.139
	Washed	36 ± 10	5.045 ± 2.223	17 ± 5	1.974 ± 0.737

^1^ Mean values (± Standard Deviation) of richness (estimated number of OTUs) and a-diversity calculates respectively using Chao1 and inverse of Simpson indexes.

## Data Availability

Raw demultiplexed sequence data were deposited in the NCBI Sequence Read Archive under the accession number SRP159168.

## Supplementary Materials

The following supporting information can be downloaded at: https://www.mdpi.com/article/10.3390/microorganisms10071381/s1, Dataset S1: Relative abundance of bacteria and fungi in all cheese core and rind samples. Compared distribution of the bacteria and fungi using all metabarcoding targets (V3–V4, V6–V8, ITS1, ITS2); Table S1: Cheese samples description and biostatistics describing cheese microflora. The richness (estimated OTUs) has been calculated with the Chao1 index, the α-diversity is evaluated with the inverse of Simpson index and the β-diversity is evaluated for the same sample, at different year using Bray–Curtis dissimilarity index, Table S2: Primers sequences for the preparation of the samples. Name of primers or adapters, target, DNA region and sequences.
