# Supplementary material for: Analysis of Microbiota Persistence in Quebec’s Terroir Cheese Using a Metabarcoding Approach
Source: Microorganisms. 2022 Jul 9;10(7):1381. doi: 10.3390/microorganisms10071381 (PMC9316450; doi:10.3390/microorganisms10071381)
Supplement: Supplementary file 1 [file microorganisms-10-01381-s001.zip › Table S1.pdf]

Table S1. Cheese samples description and biostatistics describing cheese microflora. The richness (estimated OTUs) has been calculated with the Chao1 index, the alpha-diversity is evaluated with the inverse of Simpson index and the beta-diversity is evaluated for the same sample, at different year using Bray-Curtis dissimilarity index

	Cheese				Bacteria				Fungi			
ID	Rind type	Section	Milk heat treatment	Cheese type	Sobs	Richness	α-diversity	β-diversity	Sobs	Richness	α-diversity	β-diversity
c01y15	Bloomy	core	Pasteurized	Soft	13	13.6	1.004	0.045	13	15.0	1.034	0.238
c01y18	Bloomy	core	Pasteurized	Soft	5	6.0	1.006	0.045	13	13.0	1.263	0.238
c02y15	Bloomy	core	Pasteurized	Soft	8	9.0	1.758	0.052	21	21.0	3.010	0.417
c02y18	Bloomy	core	Pasteurized	Soft	14	19.0	1.901	0.052	19	20.0	1.457	0.417
c03y15	Bloomy	core	Pasteurized	Soft	4	4.0	1.358	0.118	25	28.0	2.877	0.434
c03y18	Bloomy	core	Pasteurized	Soft	5	8.0	1.439	0.118	24	30.0	1.834	0.434
c04y15	Bloomy	core	Raw	Soft	10	10.3	1.011	0.069	17	17.8	1.749	0.286
c04y18	Bloomy	core	Raw	Soft	15	20.0	1.010	0.069	14	20.0	1.221	0.286
c06y15	Bloomy	core	Thermized	Soft	13	14.0	2.075	0.170	26	32.0	3.841	0.585
c06y18	Bloomy	core	Thermized	Soft	17	20.0	2.223	0.170	26	26.5	1.202	0.585
c18y15	Bloomy	core	Pasteurized	Semi-hard	11	11.3	1.016	-	23	30.5	2.179	-
c19y15	Bloomy	core	Raw	Semi-hard	16	17.0	2.000	-	22	22.0	2.166	-
c20y15	Bloomy	core	Pasteurized	Soft	23	25.5	1.014	-	24	25.5	1.491	-
c21y15	Bloomy	core	Pasteurized	Soft	7	8.5	1.202	-	20	20.0	3.019	-
c22y15	Bloomy	core	Pasteurized	Soft	15	51.0	1.383	-	25	26.5	1.403	-
c23y15	Bloomy	core	Raw	Soft	14	14.0	2.553	-	25	28.0	2.567	-
c07y15	Natural	core	Thermized	Semi-hard	13	23.0	2.191	0.145	17	17.0	1.298	0.431
c07y18	Natural	core	Thermized	Semi-hard	10	10.0	2.035	0.145	16	17.0	1.983	0.431
c08y15	Natural	core	Thermized	Semi-hard	10	10.5	2.040	0.269	19	20.0	3.055	0.691
c08y18	Natural	core	Thermized	Semi-hard	14	14.6	1.434	0.269	21	22.0	1.519	0.691
c24y18	Natural	core	Raw	Hard	20	24.2	1.217	-	27	27.3	3.398	-
c09y15	Washed	core	Thermized	Hard	15	20.0	1.025	0.031	24	24.0	2.220	0.540
c09y18	Washed	core	Thermized	Hard	11	13.0	1.039	0.031	22	22.0	4.651	0.540
c10y15	Washed	core	Pasteurized	Soft	14	14.8	1.926	0.052	21	21.0	2.145	0.100
c10y18	Washed	core	Pasteurized	Soft	16	21.0	2.039	0.052	28	28.0	2.120	0.100
c11y15	Washed	core	Pasteurized	Semi-hard	19	20.2	1.154	0.258	18	19.0	2.119	0.199
c11y18	Washed	core	Pasteurized	Semi-hard	16	16.0	1.485	0.258	34	40.0	1.665	0.199
c12y15	Washed	core	Thermized	Semi-hard	20	30.0	1.072	0.643	24	24.5	1.269	0.457
c12y18	Washed	core	Thermized	Semi-hard	15	15.5	2.310	0.643	28	28.3	2.414	0.457
c13y15	Washed	core	Pasteurized	Semi-hard	10	13.0	2.703	0.693	17	17.0	2.152	0.908
c13y18	Washed	core	Pasteurized	Semi-hard	23	25.0	1.066	0.693	22	32.0	1.380	0.908
c14y15	Washed	core	Thermized	Hard	9	10.5	2.305	0.281	18	18.0	3.312	0.557
c14y18	Washed	core	Thermized	Hard	7	8.0	1.504	0.281	22	22.8	2.319	0.557
c15y15	Washed	core	Pasteurized	Semi-hard	15	18.0	1.024	0.234	13	13.0	1.496	0.273
c15y18	Washed	core	Pasteurized	Semi-hard	20	30.0	1.203	0.234	33	33.3	2.445	0.273
c16y15	Washed	core	Pasteurized	Semi-hard	19	37.0	1.873	0.307	29	29.0	3.231	0.772
c16y18	Washed	core	Pasteurized	Semi-hard	10	10.0	2.095	0.307	18	21.0	1.301	0.772
c25y15	Washed	core	Raw	Hard	13	15.0	1.973	-	24	25.0	2.926	-
c26y15	Washed	core	Pasteurized	Semi-hard	13	20.5	1.018	-	20	21.0	2.578	-
c27y15	Washed	core	Thermized	Semi-hard	10	11.0	2.254	-	27	30.3	3.085	-
c28y15	Washed	core	Raw	Semi-hard	24	31.0	2.056	-	18	18.5	1.657	-
c29y15	Washed	core	Raw	Hard	22	27.3	1.869	-	19	22.0	1.505	-
c30y15	Washed	core	Thermized	Semi-hard	20	23.0	2.290	-	15	15.0	3.087	-
c31y18	Washed	core	Pasteurized	Soft	39	39.0	4.157	-	18	21.0	2.025	-
c32y18	Washed	core	Thermized	Semi-hard	22	29.5	1.759	-	23	23.0	2.974	-
c33y18	Washed	core	Thermized	Semi-hard	36	102.0	1.026	-	21	21.0	2.823	-
c34y18	Washed	core	Thermized	Hard	21	28.0	2.430	-	24	29.0	0.518	-

r01y15	Bloomy	rind	Pasteurized	Soft	-	-	-	-	9	9.0	1.003	0.461
r01y18	Bloomy	rind	Pasteurized	Soft	48	49.0	2.809	-	20	20.5	2.152	0.461
r02y15	Bloomy	rind	Pasteurized	Soft	24	29.0	2.163	0.228	15	15.3	1.540	0.268
r02y18	Bloomy	rind	Pasteurized	Soft	34	36.0	2.964	0.228	12	12.0	1.123	0.268
r03y15	Bloomy	rind	Pasteurized	Soft	26	27.0	1.392	0.510	19	20.0	2.264	0.149
r03y18	Bloomy	rind	Pasteurized	Soft	44	44.3	4.059	0.510	14	14.0	2.015	0.149
r04y15	Bloomy	rind	Raw	Soft	23	24.5	1.252	0.110	15	18.0	1.984	0.476
r04y18	Bloomy	rind	Raw	Soft	25	26.4	1.063	0.110	7	7.0	1.005	0.476
r06y15	Bloomy	rind	Thermized	Soft	24	24.2	2.468	0.769	19	19.5	2.470	0.625
r06y18	Bloomy	rind	Thermized	Soft	30	31.5	4.129	0.769	13	13.3	1.274	0.625
r18y15	Bloomy	rind	Pasteurized	Semi-hard	27	34.5	2.273	-	18	21.0	2.454	-
r19y15	Bloomy	rind	Raw	Semi-hard	31	31.5	5.476	-	19	19.0	1.579	-
r20y15	Bloomy	rind	Pasteurized	Soft	30	30.8	7.184	-	22	50.0	2.154	-
r21y15	Bloomy	rind	Pasteurized	Soft	16	17.0	1.630	-	22	25.3	1.551	-
r22y15	Bloomy	rind	Pasteurized	Soft	26	31.0	2.682	-	12	12.0	1.438	-
r23y15	Bloomy	rind	Raw	Soft	36	39.0	3.845	-	22	22.3	1.908	-
r07y15	Natural	rind	Thermized	Semi-hard	22	25.3	2.808	0.243	11	14.0	1.444	0.237
r07y18	Natural	rind	Thermized	Semi-hard	16	22.0	2.363	0.243	10	10.5	1.235	0.237
r08y15	Natural	rind	Thermized	Semi-hard	25	28.3	2.324	0.431	17	17.0	1.737	0.531
r08y18	Natural	rind	Thermized	Semi-hard	30	33.8	3.067	0.431	14	14.5	1.736	0.531
r24y18	Natural	rind	Raw	Hard	34	62.0	3.095	-	26	36.0	4.042	-
r09y15	Washed	rind	Thermized	Hard	31	31.0	7.282	0.501	16	16.5	2.807	0.537
r09y18	Washed	rind	Thermized	Hard	34	36.5	2.817	0.501	19	19.5	2.886	0.537
r10y15	Washed	rind	Pasteurized	Soft	21	22.0	4.140	0.391	16	16.0	1.745	0.148
r10y18	Washed	rind	Pasteurized	Soft	24	25.0	1.996	0.391	14	14.0	1.950	0.148
r11y15	Washed	rind	Pasteurized	Semi-hard	34	34.8	4.412	0.404	14	15.0	2.300	0.384
r11y18	Washed	rind	Pasteurized	Semi-hard	35	40.0	3.059	0.404	21	22.0	2.170	0.384
r12y15	Washed	rind	Thermized	Semi-hard	26	26.0	4.446	0.958	17	17.5	1.557	0.300
r12y18	Washed	rind	Thermized	Semi-hard	34	37.3	4.377	0.958	26	26.0	1.402	0.300
r13y15	Washed	rind	Pasteurized	Semi-hard	24	24.8	5.957	0.952	11	11.0	1.381	0.118
r13y18	Washed	rind	Pasteurized	Semi-hard	29	29.0	5.429	0.952	11	11.0	1.364	0.118
r14y15	Washed	rind	Thermized	Hard	33	33.5	5.096	0.741	10	10.0	1.277	0.579
r14y18	Washed	rind	Thermized	Hard	25	25.8	2.237	0.741	23	24.5	3.870	0.579
r15y15	Washed	rind	Pasteurized	Semi-hard	-	-	-	-	14	17.0	1.740	0.320
r15y18	Washed	rind	Pasteurized	Semi-hard	32	34.5	6.207	-	13	14.5	1.353	0.320
r16y15	Washed	rind	Pasteurized	Semi-hard	34	44.0	4.860	0.573	11	14.0	1.499	0.706
r16y18	Washed	rind	Pasteurized	Semi-hard	42	45.3	3.301	0.573	24	24.0	1.911	0.706
r25y15	Washed	rind	Raw	Hard	32	33.5	4.386	-	19	19.0	3.909	-
r26y15	Washed	rind	Pasteurized	Semi-hard	31	41.0	6.653	-	13	13.0	2.545	-
r27y15	Washed	rind	Thermized	Semi-hard	39	40.7	8.269	-	17	23.0	1.184	-
r28y15	Washed	rind	Raw	Semi-hard	38	48.0	10.969	-	12	12.0	1.453	-
r29y15	Washed	rind	Raw	Hard	40	54.0	6.693	-	21	22.5	1.844	-
r30y15	Washed	rind	Thermized	Semi-hard	38	41.3	4.450	-	10	10.5	1.534	-
r31y18	Washed	rind	Pasteurized	Soft	16	16.0	1.290	-	15	15.0	1.219	-
r32y18	Washed	rind	Thermized	Semi-hard	44	46.5	8.732	-	11	11.5	2.066	-
r33y18	Washed	rind	Thermized	Semi-hard	33	33.0	4.953	-	11	12.0	2.337	-
r34y18	Washed	rind	Thermized	Hard	41	59.0	4.121	-	20	30.0	2.025	-
