# Supplementary material for: Analysis of Microbiota Persistence in Quebec’s Terroir Cheese Using a Metabarcoding Approach
Source: Microorganisms. 2022 Jul 9;10(7):1381. doi: 10.3390/microorganisms10071381 (PMC9316450; doi:10.3390/microorganisms10071381)
Supplement: Supplementary file 1 [file microorganisms-10-01381-s001.zip › Table S2.pdf]

Table S2. Primers sequences for the preparation of the samples. Name of primers or adapters, target, DNA region and sequences.

Primers/adapters	Target	Region	Sequences
347F (a)	Bacteria	V3-V4	GGAGGCAGCAGTRRGGAAT
803R (a)	Bacteria	V3-V4	CTACCRGGGTATCTAATCC
341F (b)	Bacteria	V3-V4	CCTACGGGNGGCWGCAG
805R (b)	Bacteria	V3-V4	GACTACHVGGGTATCTAATCC
969F	Bacteria	V6-V8	ACGCGHNRAACCTTACC
1406R	Bacteria	V6-V8	ACGGGCRGTGWGTRCAA
ITS1Fngs	Fungi	ITS1	GGTCATTTAGAGGAAGTAA
ITS2	Fungi	ITS1	GCTGCGTTCTTCATCGATGC
ITS3tagmix1	Fungi	ITS2	TAGACTCGTCATCGATGAAGAACGCAG
ITS3tagmix2	Fungi	ITS2	TAGACTCGTCAACGATGAAGAACGCAG
ITS3tagmix3	Fungi	ITS2	TAGACTCGTCACCGATGAAGAACGCAG
ITS3tagmix4	Fungi	ITS2	TAGACTCGTCATCGATGAAGAACGTAG
ITS3tagmix5	Fungi	ITS2	TAGACTCGTCATCGATGAAGAACGTGG
ITS4ngs	Fungi	ITS2	TTCCTSCGCTTATTGATATGC
Forward adapter	-	-	ACACTCTTTCCCTACACGACGCTCTTCCGATCT
Reverse adapter	-	-	GTGACTGGAGTTCAGACGTGTGCTCTTCCGATCT

(a) Primers used in 2015; (b) Primers used in 2018.
